# cAMP/Protein Kinase A Signaling Inhibits Dlx5 Expression via Activation of CREB and Subsequent C/EBPβ Induction in 3T3-L1 Preadipocytes

**DOI:** 10.3390/ijms19103161

**Published:** 2018-10-14

**Authors:** Hye-Lim Lee, Abdul S. Qadir, Hyun-Jung Park, Eunkyung Chung, Yun-Sil Lee, Kyung Mi Woo, Hyun-Mo Ryoo, Hyun Jeong Kim, Jeong-Hwa Baek

**Affiliations:** 1 Department of Molecular Genetics, School of Dentistry and Dental Research Institute, Seoul National University, Seoul 08826, Korea; hyelim.lee.lhl@gmail.com (H.-L.L.); abdul.syed@northwestern.edu (A.S.Q.); in2010@snu.ac.kr (H.-J.P.); yunlee@snu.ac.kr (Y.-S.L.); kmwoo@snu.ac.kr (K.M.W.); hmryoo@snu.ac.kr (H.-M.R.); 2 Department of Anatomy & Neurobiology, University of California, Irvine, CA 92697, USA; 3 Division of Hematology/Oncology, Feinberg School of Medicine, Northwestern University, Chicago, IL 60611, USA; 4 Research Center, SMD Solution Co., Ltd., Seoul 03080, Korea; chungek@hanmail.net; 5 Department of Dental Anesthesiology, School of Dentistry, Seoul National University, Seoul 03080, Korea; dentane@snu.ac.kr

**Keywords:** CREB, C/EBPβ, IBMX, Dlx5, adipocyte

## Abstract

Distal-less homeobox 5 (Dlx5) is a negative regulator of adipogenesis. Dlx5 expression is decreased by adipogenic stimuli, but the mechanisms of Dlx5 downregulation by adipogenic stimuli have not yet been determined. Here, we tested the impact of cAMP/PKA (protein kinase A) signaling induced by 3-isobutyl-1 methyl xanthine (IBMX), forskolin, and 8-CPT-cAMP on the expression of Dlx5 in 3T3-L1 preadipocytes. Significant downregulation of Dlx5 mRNA expression and protein production levels were observed via cAMP/PKA-dependent signaling. Forced expression of cAMP-responsive element-binding protein (CREB) and CCAAT/enhancer-binding protein β (C/EBPβ) was sufficient for downregulation of Dlx5 expression and revealed that CREB functions upstream of C/EBPβ. In addition, C/EBPβ knockdown by siRNA rescued Dlx5 expression in IBMX-treated 3T3-L1 preadipocytes. Luciferase assays using a Dlx5-luc-2935 reporter construct demonstrated the requirement of the *Dlx5* promoter region, ranging from −774 to −95 bp that contains two putative C/EBPβ binding elements (site-1: −517 to −510 bp and site-2: −164 to −157 bp), in the suppression of Dlx5 transcription. Consequently, chromatin immunoprecipitation analysis confirmed the importance of site-1, but not site-2, in C/EBPβ binding and transcriptional suppression of Dlx5. In conclusion, we elucidated the underling mechanism of Dlx5 downregulation in IBMX-induced adipogenesis. IBMX activated cAMP/PKA/CREB signaling and subsequently upregulated C/EBPβ, which binds to the *Dlx5* promoter to suppress Dlx5 transcription.

## 1. Introduction

Obesity has become a rising issue related with various health problems in modern society [1]. Obesity is a medical condition of excessive fat mass that is caused by an increase in fat cell number and size. Fat tissue is mainly composed of adipocytes that play an important role in energy metabolism; therefore, studying the molecular mechanisms of adipogenic differentiation and its regulatory factors might produce clues to the etiology of obesity and other metabolic diseases.

Distal-less homeobox 5 (Dlx5) is a well-known transcription factor involved in bone development. Dlx5 upregulates osteogenesis by activating Runx2, a master transcriptional regulator for osteoblast differentiation [2]. Bone morphogenetic protein 2 (Bmp2) induces Dlx5 expression, which in turn binds to the *Runx2* promoter and enhances Runx2 expression. Dlx5 also mediates Bmp2-induced expression of Sp7, another key transcription factor for osteoblastic differentiation [3]. We previously demonstrated that Dlx5 inhibits adipogenic differentiation through downregulation of peroxisome proliferator-activated receptor γ (PPARγ) and that Dlx5 expression is downregulated by adipogenic stimuli [4]. We also reported that, among the many constituents responsible for adipogenesis induction, insulin downregulates Dlx5 expression by inducing miR-124 [5]. However, the mechanism by which Dlx5 expression is inhibited has not yet been characterized.

3T3-L1 preadipocytes are a well-established in vitro cell culture model to study adipogenic differentiation [6]. 3T3-L1 preadipocytes are converted to adipocytes by an induction mixture containing 3-isobutyl-1-methylxanthine (IBMX), insulin, dexamethasone, and indomethacin [7] that causes induction of transcriptional regulators such as CCAAT/enhancer binding protein (C/EBP) β, δ, and α and PPARγ, leading to differentiation of preadipocytes toward mature adipocytes [8]. The cAMP-response element binding protein (CREB) is another key transcriptional regulator of adipogenesis [9,10,11] that is constitutively expressed in preadipocytes and throughout the differentiation process toward mature adipocytes [10,12]. CREB is activated via protein kinase A (PKA)-mediated phosphorylation, and activated CREB binds to the cAMP response element (CRE) in the promoters of several adipocyte-specific genes to regulate their transcription [9,10].

In the early stages of adipogenesis, accumulation of cAMP stimulates the activation and expression of C/EBPβ and δ [13,14]. It has been reported that during adipogenic differentiation, CREB-induced C/EBPβ promotes activation of C/EBPα and PPARγ, which are the master transcriptional regulators of adipogenesis [10,11]. C/EBPα and PPARγ then induce the transcription of the set of genes that gives rise to the adipocyte phenotype [9].

In the present study, we characterized the underling mechanisms of Dlx5 downregulation in IBMX-induced adipogenesis. IBMX activated cAMP/PKA/CREB signaling and subsequently upregulated C/EBPβ, which binds to the promoter region of Dlx5 and suppresses Dlx5 transcription.

## 2. Results

### 2.1. Activation of cAMP/PKA Signaling Inhibits Distal-Less Homeobox 5 (Dlx5) Expression in 3T3-L1 Preadipocytes

**Figure 1:**
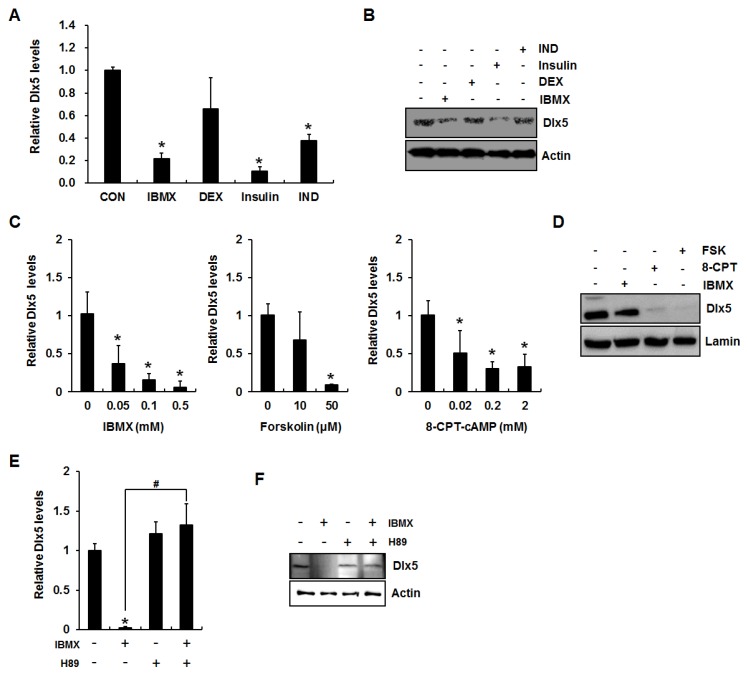
Activation of cAMP/PKA (protein kinase A) signaling inhibits distal-less homeobox 5 (Dlx5) expression in 3T3-L1 preadipocytes. (**A**) PKA activation suppresses Dlx5 expression during adipogenesis. Two-day post confluent 3T3-L1 cells were incubated in Dulbecco’s modified Eagle’s medium (DMEM) supplemented with 10% fetal bovine serum (FBS) with indicated concentration of 0.5 mM 3-isobutyl-1-methylxanthine (IBMX), 0.1 µM dexamethasone (DEX), 10 µg/mL insulin, and 50 µM indomethacin (IND) for 24 h, and Dlx5 mRNA levels were determined by quantitative RT-PCR. (**B**) Dlx5 protein level was determined by Western blot. (**C**) Dlx5 mRNA level was determined in the cells treated with various concentrations of IBMX, forskolin, or 8-CPT-cAMP for 24 h. (**D**) Dlx5 protein level after 24 h treatment with 0.5 mM IBMX, 2 mM 8-CPT-cAMP (8-CPT), or 50 µM forskolin (FSK) was determined by Western blot. In IBMX-induced adipogenesis, inhibition of PKA activation by 50 µM H89 completely rescued Dlx5 mRNA expression (**E**) and protein (**F**) levels. Data represent the mean ± SD (standard deviation) (* *p* < 0.05, compared to control, CON; # *p* < 0.05 for the indicated pairs).

Because our previous study demonstrated that an adipogenesis induction mixture suppresses Dlx5 expression [4], we first examined the effect of each component of the induction mix on the expression of Dlx5. 3T3-L1 cells were incubated for 24 h in the presence of each adipogenic component (0.5 mM IBMX, 0.1 µM dexamethasone, 10 µg/mL insulin, or 50 µM indomethacin), and qRT-PCR and Western blot analyses were performed to detect Dlx5 mRNA and protein expression, respectively. Consistent with the results from a previous report [5], insulin strongly suppressed Dlx5 expression (Figure 1A,B). In addition, IBMX significantly downregulated Dlx5 mRNA and protein expression levels (Figure 1A,B). Therefore, we more closely examined the effect of IBMX on Dlx5 expression in this study.

IBMX is an inhibitor of cAMP phosphodiesterase, resulting in accumulation of cAMP and activation of PKA. Therefore, we next examined whether accumulation of cAMP exerts a Dlx5 inhibitory effect using IBMX, a cell-permeable cAMP analogue (8-CPT-cAMP), or the adenylate cyclase activator forskolin [8,15,16]. 3T3-L1 cells were incubated for 24 h in the presence of IBMX (0.05, 0.1, or 0.5 mM), forskolin (10 or 50 µM), or 8-CPT-cAMP (0.02, 0.2, or 2 mM), followed by qRT-PCR of the *Dlx5* gene. qRT-PCR results demonstrated that all the reagents inducing cAMP accumulation or reagents mimicking cAMP action significantly decreased Dlx5 mRNA level (Figure 1C). Western blot analysis confirmed the inhibitory effects of IBMX (0.5 mM), forskolin (50 µM), and 8-CPT-cAMP (2 mM) on Dlx5 expression (Figure 1D). IBMX is most often used in adipogenic stimuli mixtures to induce adipogenesis in in vitro models and is well known for cAMP activation; therefore, further experiments were carried out using IBMX. To further confirm involvement of the cAMP/PKA signaling cascade in IBMX-induced reduction of Dlx5 expression in 3T3-L1 cells, we applied the widely-used PKA inhibitor H89 (20 µM) in the presence of IBMX. As expected, inhibition of PKA activation by H89 fully rescued the Dlx5 mRNA and protein expression levels in the presence of IBMX (Figure 1E,F). These results suggest that cAMP accumulation and subsequent activation of PKA are key elements for Dlx5 inhibition during IBMX-induced adipocyte differentiation in 3T3-L1 cells. 

### 2.2. CREB Mediates cAMP/PKA Signaling-Induced Dlx5 Suppression via Upregulation of C/EBPβ

**Figure 2:**
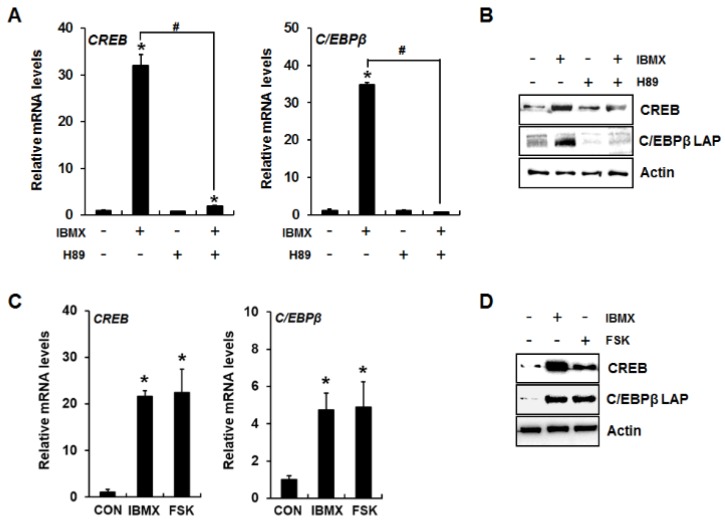
Induction of CREB and C/EBPβ expression in IBMX-treated 3T3-L1 preadipocytes via cAMP/PKA signaling. IBMX- or forskolin-induced changes in CREB and C/EBPβ expression were examined at the mRNA (**A**,**C**) and protein (**B**,**D**) levels. Two days after confluence, 3T3-L1 preadipocytes were incubated with the indicated reagent for 24 h. Then, CREB and C/EBPβ mRNA and protein levels were analyzed by quantitative RT-PCR and Western blot, respectively. Data represent the mean ± SD (* *p* < 0.05, compared to control, CON, and # *p* < 0.05, compared between IBMX-treated groups in the presence or absence of H89). IBMX, 0.5 mM; H89, 20 µM; FSK (forskolin), 50 µM.

It has been demonstrated that cAMP/PKA signaling increases the activity and expression of CREB and C/EBPβ [17]. Therefore, we examined whether activation of cAMP/PKA signaling by IBMX or forskolin increases CREB and C/EBPβ mRNA and protein expression levels. IBMX significantly increased CREB and C/EBPβ mRNA and protein expression levels, which were blocked by the PKA inhibitor H89 (Figure 2A,B). Forskolin treatment also induced expression of CREB and C/EBPβ (Figure 2C,D), indicating that PKA activation lead to enhanced expression of the CREB and C/EBPβ transcription factors.

**Figure 3:**
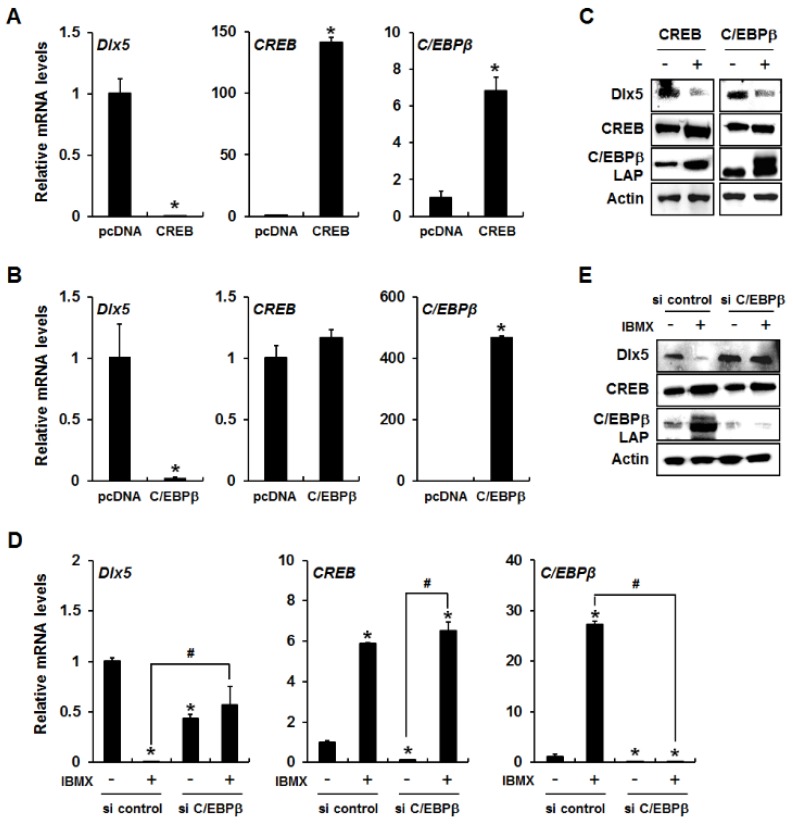
IBMX-induced Dlx5 suppression via upregulation of CREB and subsequent C/EBPβ expression. Overexpression of CREB or C/EBPβ inhibits Dlx5 expression in 3T3-L1 cells. 3T3-L1 cells were transfected with either control (pcDNA), CREB, or C/EBPβ expression vectors and allowed to express for 24 h. Then, the mRNA and protein levels of Dlx5, CREB, and C/EBPβ were analyzed by quantitative RT-PCR (**A**,**B**) and Western blot (**C**), respectively. In IBMX-treated 3T3-L1 preadipocytes, knockdown of C/EBPβ by siRNA rescued the expression of Dlx5 at both the mRNA (**D**) and protein (**E**) levels. C/EBPβ knockdown did not exert any effect on IBMX-induced CREB expression. Data represent the mean ± SD (* *p* < 0.05, compared to control, pcDNA, and si control, # *p* < 0.05, compared between IBMX-treated groups in the presence or absence of suppression of C/EBPβ by siRNA).

To determine whether CREB and C/EBPβ play a role in cAMP/PKA signaling-induced downregulation of Dlx5, we examined Dlx5 expression levels after forced expression of CREB or C/EBPβ. As shown in Figure 3A, a significant reduction of Dlx5 mRNA was observed in CREB-overexpressed 3T3-L1 cells with consistent induction of C/EBPβ mRNA as a downstream target of CREB. In addition, forced expression of C/EBPβ alone in 3T3-L1 cells clearly inhibited Dlx5 mRNA expression. However, C/EBPβ overexpression did not show any change in CREB mRNA expression, indicating that CREB functions upstream of C/EBPβ (Figure 3B). The downregulation of Dlx5 induced by CREB and C/EBPβ overexpression was confirmed in protein level as well (Figure 3C). Collectively, these results suggest that induction of C/EBPβ is required for inhibition of Dlx5 expression.

To further confirm that C/EBPβ is required for inhibition of Dlx5 expression, we knocked down C/EBPβ in 3T3-L1 cells using C/EBPβ siRNA and incubated the cells for 24 h in the presence or absence of IBMX. C/EBPβ silencing efficiency was confirmed by qRT-PCR and Western blot analysis (Figure 3D,E). C/EBPβ knockdown did not exert any effect on IBMX-induced CREB expression, further supporting that C/EBPβ works downstream of CREB (Figure 3D,E). When comparing the Dlx5 expression in control siRNA-transfected cells versus C/EBPβ siRNA-transfected cells in the absence of IBMX treatment, C/EBPβ knockdown significantly reduced Dlx5 mRNA levels but slightly increased Dlx5 protein levels (Figure 3D,E). However, when comparing the Dlx5 expression in vehicle-treated cells versus IBMX-treated cells, C/EBPβ knockdown fully blocked IBMX-induced downregulation of Dlx5 expression (Figure 3D,E). These results suggest that C/EBPβ is a key regulator in cAMP/PKA signaling-mediated Dlx5 downregulation, and that CREB downregulates Dlx5 expression via upregulation of C/EBPβ during IBMX-induced adipogenesis of 3T3-L1 cells.

### 2.3. C/EBPβ Binds to the Dlx5 Promoter and Suppresses Dlx5 Transcription

**Figure 4:**
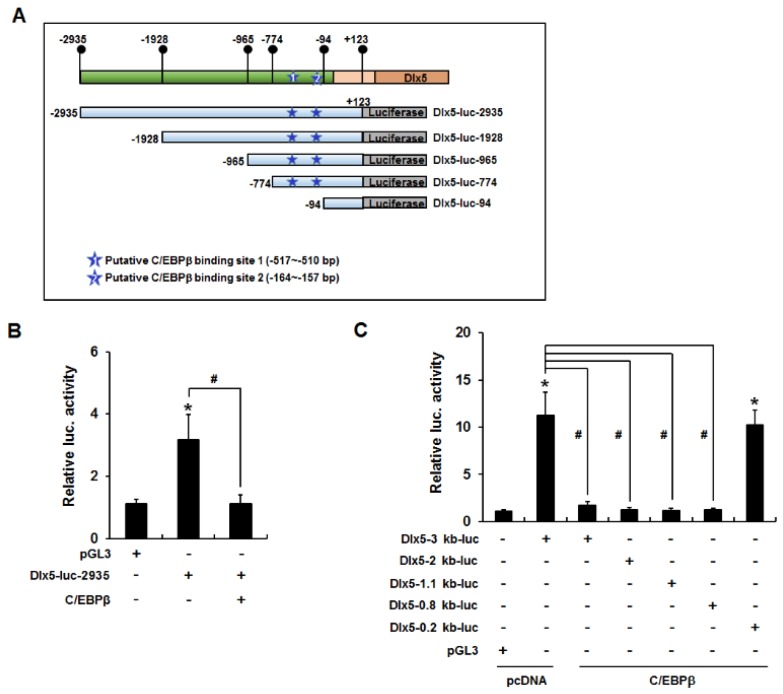
C/EBPβ binding to the −774 to −95 bp *Dlx5* promoter region is necessary for C/EBPβ to suppress Dlx5 transcription. (**A**) A Dlx5-luc-2935 reporter construct that contains the mouse *Dlx5* promoter sequence (−2935 to +123 bp) and its serial deletion constructs, Dlx5-luc-1928, Dlx5-luc-965, Dlx5-luc-774, and Dlx5-luc-94. Putative C/EBPβ binding elements (site-1: −517 to −510 bp, site-2: −164 to −157 bp) are indicated as predicted by the transcription factor binding site search tools TESS and PROMO. (**B**) Luciferase reporter assay using the Dlx5-luc-2935 construct and the C/EBPβ expression vector displayed suppression of Dlx5 promoter activity similar to the inhibitory effect on Dlx5 mRNA expression. (**C**) Inhibition of Dlx5 promoter activity was maintained in cells containing the Dlx5-luc-2935, Dlx5-luc-1928, Dlx5-luc-965, or Dlx5-luc-774 reporter, whereas luciferase activity was recovered to control level in the Dlx5-luc-94 reporter. Data represent the mean ± SD (* *p* < 0.05, compared to pGL3, # *p* < 0.05, significant difference in the indicated pairs).

Because C/EBPβ decreased Dlx5 mRNA and protein levels, we hypothesized that C/EBPβ reduces Dlx5 expression by inhibiting Dlx5 transcription. To test this hypothesis, we performed luciferase reporter assays using a Dlx5-luc-2935 reporter construct that contains the mouse *Dlx5* promoter sequence (−2935 to +123 bp). 3T3-L1 cells were transiently transfected with Dlx5-luc-2935 and/or the C/EBPβ expression vector and incubated in growth medium for 24 h. *Renilla* luciferase was applied as a control for normalization of transfection efficiency. Similar to the inhibitory effect on Dlx5 mRNA expression, C/EBPβ overexpression clearly suppressed Dlx5 promoter activity (Figure 4B). To narrow the *Dlx5* promoter region involved in the C/EBPβ-mediated inhibition of Dlx5 expression, we repeated luciferase reporter assays using the serial deletion constructs depicted in Figure 4A. The inhibitory effect of C/EBPβ was maintained in cells that contained the Dlx5-luc-2935, Dlx5-luc-1928, Dlx5-luc-965, or Dlx5-luc-774 reporter, whereas luciferase activity was recovered to control level in the cells containing the Dlx5-luc-94 reporter in the presence of C/EBPβ overexpression (Figure 4C). These results suggest that the *Dlx5* promoter region from −774 to −95 bp is necessary for C/EBPβ to suppress Dlx5 transcription.

**Figure 5:**
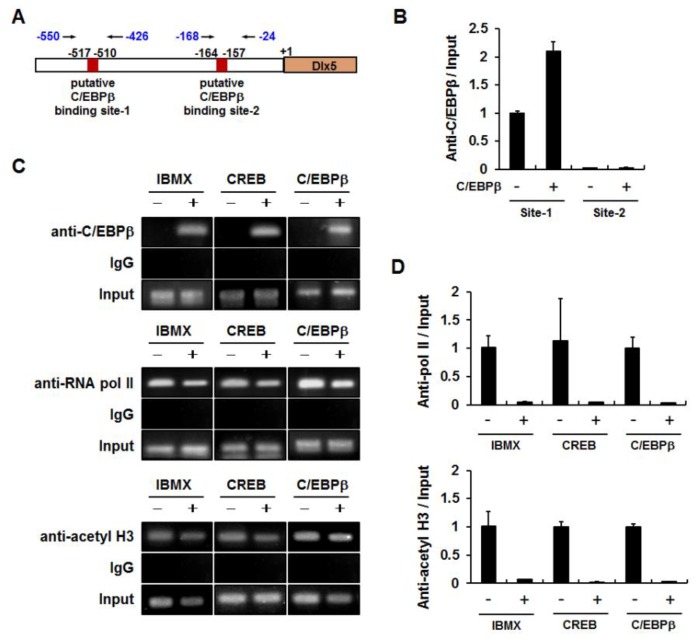
Increased binding of C/EBPβ to the *Dlx5* promoter region (−517 to −510 bp) is correlated with reduced binding of RNA polymerase II and acetyl histone H3 to the *Dlx5* promoter. (**A**) The location of PCR-amplified regions in the *Dlx5* promoter and the putative C/EBPβ binding site-1 (−517 to −510 bp) and site-2 (−164 to −157 bp) are depicted. (**B**) Quantitative PCR of the *Dlx5* promoter regions demonstrated specific binding to C/EBPβ binding site-1, but not to C/EBPβ binding site-2. The relative ratio of C/EBPβ antibody bound fraction to input in C/EBPβ binding site-2 was less than 3% of that at C/EBPβ binding site-1. The nuclear extracts were selected by immunoprecipitation with anti-C/EBPβ antibodies. (**C**,**D**) Immunoprecipitation with anti-C/EBPβ antibodies confirmed increased binding of C/EBPβ to the C/EBPβ binding site-1 in response to IBMX and CREB or C/EBPβ expression. Decreased binding of RNA polymerase II (RNA pol II) and acetyl histone H3 (acetyl H3) to the *Dlx5* promoter is shown by immunoprecipitation with anti-RNA pol II and anti-acetyl H3 antibodies, respectively. Immunoprecipitation with the IgG control supported chromatin immunoprecipitation (ChIP) specificity. Quantitative ChIP of RNA pol II and acetyl H3 data were normalized to the input and presented as the relative values to vehicle-treated or pcDNA-transfected control cells (**D**).

We then investigated whether the *Dlx5* promoter region (−774 to −95 bp) contains putative C/EBPβ binding elements. As depicted in Figure 4A, there were two putative C/EBPβ binding elements (site-1: −517 to −510 bp and site-2: −164 to −157 bp), which were predicted as putative C/EBPβ binding sites based on the transcription factor binding site search tools, TESS and PROMO. Therefore, we performed ChIP assays to determine whether C/EBPβ interacts with these putative binding sites. The nuclear extracts from cells transiently transfected with pcDNA or C/EBPβ expression plasmids were used for immunoprecipitation with anti-C/EBPβ antibodies. Quantitative PCR of the *Dlx5* promoter regions demonstrated that the relative ratio of C/EBPβ antibody bound fraction to input in the C/EBPβ binding site-2 was less than 3% of that of the C/EBPβ binding site-1 (Figure 5B). Furthermore, overexpression of C/EBPβ increased the C/EBPβ antibody bound fraction to the C/EBPβ binding site-1 but not the C/EBPβ binding site-2. Therefore, we ruled out the putative C/EBPβ binding site-2 and performed further ChIP experiments targeting the C/EBPβ binding site-1. The 3T3-L1 cells were treated with IBMX for 24 h or transiently transfected with CREB or C/EBPβ expression plasmid. PCR amplification of the *Dlx5* promoter region encompassing the C/EBPβ binding site-1 demonstrated that IBMX treatment or overexpression of CREB or C/EBPβ clearly increased C/EBPβ binding to the site-1 *Dlx5* promoter region (Figure 5C). ChIP specificity was supported by showing that PCR products were not detected in the samples immunoprecipitated with IgG control.

Because Dlx5 promoter activity was suppressed by C/EBPβ (Figure 4B,C), we examined whether the binding of RNA pol II to the *Dlx5* promoter region was also affected by C/EBPβ. ChIP assays with anti-pol II antibody demonstrated that IBMX, CREB, and C/EBPβ decreased the binding of RNA pol II to the *Dlx5* promoter (Figure 5C,D). We also examined whether decreased Dlx5 expression correlated with reduced histone acetylation level in the *Dlx5* promoter. ChIP assays with anti-acetyl histone H3 antibody demonstrated that IBMX, CREB, and C/EBPβ reduced the histone H3 acetylation level in the *Dlx5* promoter (Figure 5C,D). These results suggest that enhanced binding of C/EBPβ to the *Dlx5* promoter region plays an important role in C/EBPβ-mediated suppression of Dlx5 transcription.

## 3. Discussion

Dlx5 plays important and evolutionally conserved roles in the development of mineralized tissues by promoting osteogenic differentiation [18,19]. In addition, Dlx5 acts as a negative regulator of adipogenic differentiation and downregulation of Dlx5 is necessary for proceeding adipocyte differentiation [4]. Dlx5 expression is rapidly downregulated by adipogenic stimuli, among which insulin inhibits Dlx5 expression through induction of miR-124, a Dlx5 targeting miRNA [5]. However, the mechanism of Dlx5 downregulation with other components of adipogenic stimuli during adipogenesis have not yet been elucidated. 

In the present study, we demonstrated that (i) in 3T3-L1 preadipocytes, activation of cAMP/PKA signaling by IBMX, forskolin, and 8-CPT-cAMP suppressed Dlx5 expression; (ii) activation of cAMP/PKA signaling induced C/EBPβ expression in a CREB-dependent manner; (iii) C/EBPβ induction is sufficient to inhibit Dlx5 expression; and (iv) C/EBPβ binds to the *Dlx5* promoter to suppress Dlx5 transcription.

Forskolin induces cAMP accumulation by activating the adenylate cyclase. IBMX, prostacyclin, and 8-CPT-cAMP are well known adipogenic inducers through the cAMP induction and PKA activation pathway [8,15,20]. When 3T3-L1 preadipocytes were induced with IBMX, forskolin, or 8-CPT-cAMP, Dlx5 expression was suppressed, suggesting that the suppressive effects of IBMX, forskolin, and 8-CPT-cAMP on Dlx5 expression may be ascribed to cAMP-dependent signaling pathways. Although PKA is the major downstream molecule activated by cAMP, it is not clearly defined whether the cAMP-PKA pathways play an essential role in adipogenic differentiation in 3T3-L1 cells [17,21,22]. Therefore, we further analyzed whether PKA pathway involvement is necessary to inhibit Dlx5 expression by cAMP. In the presence of the PKA inhibitor H89, IBMX could not inhibit Dlx5 expression, indicating that the cAMP/PKA signaling pathway is involved in Dlx5 regulation.

It is well established that PKA is directly involved in CREB activation by inducing phosphorylation of Ser133, which is necessary for transcriptional activation [10]. CREB has been shown to regulate initiation of adipocyte differentiation by inducing the expression of C/EBPβ [9,10]. In the present study, IBMX induced the expression of both CREB and C/EBPβ, which was blocked by the PKA inhibitor H89. In addition, we confirmed that CREB increases C/EBPβ expression. Therefore, the activation and induction of CREB and the ensuing induction of C/EBPβ and inhibition of Dlx5 expression appear to depend on the cAMP-PKA signaling pathway.

C/EBPβ is an important regulator of mitotic clonal expansion of preadipocytes as well as the adipogenic differentiation [23]. The activation of C/EBPβ triggers C/EBPα and PPARγ gene expression, and C/EBPα and PPARγ activate the transcription of the set of genes that give rise to the adipocyte phenotype. Although the majority of studies describe C/EBPβ as a transcriptional activator, some reports have demonstrated that C/EBPβ downregulates the expression levels of some target genes. It has been reported that C/EBPβ binds to the CD200R1 promoter and inhibits CD200R1 expression in microglial cells [24]. C/EBPβ also binds to the promoter region and inhibits the expression of the human hepatic stimulator substance gene, which acts as a hepatotropic growth factor and promotes liver regeneration [25]. In the present study, sequential changes in the expression levels of Dlx5 and C/EBPβ lead to the hypothesis that inhibition of Dlx5 expression is due to increased C/EBPβ. The experimental results from the overexpression of CREB and C/EBPβ support this hypothesis. Overexpression of CREB or C/EBPβ suppressed the expression levels of Dlx5 mRNA and protein. Conversely, C/EBPβ knockdown completely blocked IBMX-induced suppression of Dlx5 mRNA and protein expression. These findings imply that decreased Dlx5 expression is ascribed to upregulation of C/EBPβ expression. Interestingly, C/EBPβ knockdown itself decreased basal Dlx5 mRNA level but increased the basal Dlx5 protein level (Figure 3D,E). Admittedly, we don’t have a good explanation for this discrepancy in basal expression levels of mRNA and protein in C/EBPβ-silenced cells. However, considering that cAMP/PKA-induced activation of CREB and subsequent upregulation of C/EBPβ is strongly induced by IBMX, we believe that it may be reasonable to compare the Dlx5 expression level in following pairs: (i) vehicle-treated, control siRNA-transfected cells versus IBMX-treated, control siRNA-transfected cells, and (ii) vehicle-treated, C/EBPβ-silenced cells versus IBMX-treated, C/EBPβ-silenced cells (Figure 3D,E). Our results demonstrated that IBMX-induced downregulation of Dlx5 mRNA and protein levels was completely blocked in C/EBPβ-silenced cells whereas IBMX clearly suppressed Dlx5 mRNA and protein expression in control siRNA-transfected cells. Therefore, we conclude that C/EBPβ mediates IBMX-induced downregulation of Dlx5 in 3T3-L1 cells.

Dlx5 promoter reporter assays further support the transcriptional repressor role of C/EBPβ by showing that C/EBPβ suppresses Dlx5 promoter activity in a −774 to −95 bp *Dlx5* promoter region-dependent manner. Subsequently, Dlx5 promoter reporter assay results were further supported by ChIP experiments, which showed that IBMX treatment or overexpression of CREB and C/EBPβ in 3T3-L1 cells increased the binding of C/EBPβ but decreased that of RNA pol II and acetyl histone H3 to the *Dlx5* promoter region −550 to −426 bp, which encompasses a putative C/EBPβ binding site (−517 to −510 bp). These results suggest that enhanced binding of C/EBPβ to the *Dlx5* promoter leads to histone modification status for transcriptional repression and reduces recruitment of RNA pol II. However, further studies are necessary to confirm the direct binding of C/EBPβ to the *Dlx5* promoter by repeating Dlx5 promoter reporter assays using a construct with mutations in the putative C/EBPβ binding site (−517 to −510 bp) or by performing electrophoretic mobility shift assays.

Taken together, these results demonstrate that the adipogenic differentiation inducer IBMX suppresses Dlx5 transcription in 3T3-L1 preadipocytes in a cAMP/PKA/CREB/C/EBPβ-dependent manner. Considering the negative regulatory role of Dlx5 in adipogenesis, this finding suggests that Dlx5 inhibition contributes to the enhancement of adipogenesis triggered by C/EBPβ expression during adipogenic differentiation.

## 4. Materials and Methods

### 4.1. Materials and Cell Culture

IBMX, forskolin, 8-CPT-cAMP, and H89 were purchased from Sigma (St Louis, MO, USA). Anti-Dlx5 antibody was purchased from Millipore (Billerica, MA, USA). Anti-RNA polymerase II (pol II), anti-actin antibody, and HRP-conjugated secondary antibodies were obtained from Santa Cruz Biotechnology (Santa Cruz, CA, USA). Anti-C/EBPBβ, anti-acetyl-histone H3 (H3), and anti-CREB antibodies were obtained from Cell Signaling Technology (Danvers, MA, USA). Dulbecco’s modified Eagle’s medium (DMEM) and fetal bovine serum (FBS) were purchased from HyClone (Logan, UT, USA). 

The C/EBPβ expression vector was kindly provided by Prof. JB Kim from Seoul National University. Construction of the CREB expression vector was described previously [26].

3T3-L1 cells were cultured in DMEM supplemented with 10% FBS, 100 U/ml penicillin, and 100 µg/mL streptomycin. After 2 days of confluence, the cells were treated with the indicated reagents (IBMX, forskolin, 8-CPT-cAMP, or H89) for the indicated time periods. To induce exogenous expression of CREB or C/EBPβ, 3T3-L1 cells were transfected with expression vectors (pcDNA3.1, CREB or C/EBPβ) using lipofectamine 2000 reagent (Thermo Fisher Scientific; Waltham, MA, USA) and incubated for 24 h.

### 4.2. Quantitative Reverse Transcription-Polymerase Chain Reaction (qRT-PCR)

Total RNA isolation and qRT-PCR was performed as described previously [5]. Each sample was analyzed in triplicate and expression levels of target genes were normalized to the glyceraldehyde-3-phosphate dehydrogenase (GAPDH). Fold changes of each treatment group were calculated using normalized *C_T_* values compared to the control. The sequences (5′→3′) of forward and reverse primers for each gene were as follows: *Dlx5*, TCT CTA GGA CTG ACG CAA ACA and GTT ACA CGC CAT AGG GTC GC; *CREB*, AGC TGC CAC TCA GCC GGG TA and TGG TGC TCG TGG GTG CTG TG; *C/EBPβ*, GAC TTT ATG GGC AGC TTT GC and GGC TTT GTC TCT GCT TTT GC; and *GAPDH*, TCA ATG ACA ACT TTG TCA AGC and CCA GGG TTT CTT ACT CCT TGG.

### 4.3. Western Blot Analysis

Whole cell extracts were prepared using PRO-PREP^TM^ Protein Extraction Solution (iNtRON Biotechnology, Sungnam, Korea) according to the manufacturer’s instructions. Western blot analysis was then performed as described previously [4].

### 4.4. C/EBPβ Knockdown by Small Interfering RNA (siRNA)

C/EBPβ siRNA and control siRNA (ON-TARGET*plus* SMART pool, L-043110-00-0005 and Non-targeting siRNA, D-001810-10-05) were purchased from Dharmacon (Chicago, IL, USA), and transient transfection of siRNA was performed using Dharmafect (Dharmacon).

### 4.5. Luciferase Reporter Assays

The Dlx5 promoter reporter plasmid, containing the −2935 bp to +123 bp mouse *Dlx5* promoter region sequence upstream of the luciferase gene of the pGL3-basic vector (Dlx5-luc-2935), and serial deletion constructs (Dlx5-luc-1928, Dlx5-luc-965, Dlx5-luc-774, and Dlx5-luc-94) were kindly provided by Prof. HM Ryoo from Seoul National University. To measure luciferase activity, 3T3-L1 cells were plated at an initial density of 1 × 10^4^ cells per well into 96-well plates. After overnight culture, the cells were transiently transfected with the indicated plasmid using lipofectamine 2000. In each transfection, 0.2 µg of expression vector (C/EBPβ or pcDNA) and 0.2 µg of reporter plasmids were used. The *Renilla* luciferase-expressing plasmid was also added to normalize transfection efficiency. Luciferase activity was measured after 24 h-incubation using a Dual-Glo luciferase assay kit (Promega, Madison, WI, USA).

### 4.6. Chromatin Immunoprecipitation (ChIP) Assays

ChIP assays were performed as described previously using anti-C/EBPβ, anti-RNA polymerase II (RNA pol II), and anti-acetyl histone H3 (acetyl H3) antibodies [4]. DNA eluted from the immune complexes was subjected to PCR amplification of mouse *Dlx5* promoter regions containing putative C/EBPβ binding elements. The forward and reverse primer sequences are as follows (5′→3′): *Dlx5* promoter region-1 (−550 to −426 bp) CCG TCG GAG GAG GGG GGA GAG G and GCT CCG TGC TGT TTG AAG ACA ACG; and *Dlx5* promoter region-2 (−168 to −24 bp) CTC TTT AAG CAA TGC TTT GTT GTG C and GGC GCA GCA CAG CCT TGG TTA AAT C.

### 4.7. Statistical Analysis

Quantitative data are presented as the mean ± SD. Statistical analysis was performed with Student’s *t*-tests and a *p*-value less than 0.05 was considered statistically significant.
